# Imaging segmentation mechanism for rectal tumors using improved U-Net

**DOI:** 10.1186/s12880-024-01269-6

**Published:** 2024-04-23

**Authors:** Kenan Zhang, Xiaotang Yang, Yanfen Cui, Jumin Zhao, Dengao Li

**Affiliations:** 1 College of Computer Science and Technology (College of Data Science), Taiyuan University of Technology, Taiyuan, 030024 China; 2 College of Electronic Information and Optical Engineering, Taiyuan University of Technology, Taiyuan, 030024 China; 3 Key Laboratory of Big Data Fusion Analysis and Application of Shanxi Province, Taiyuan, 030024 China; 4 Intelligent Perception Engineering Technology Center of Shanxi, Taiyuan, 030024 China; 5 Department of Radiology, Shanxi Cancer Hospital, Shanxi Medical University, Taiyuan, 030013 China

**Keywords:** Semantic segmentation, U-Net, Rectal cancer, MR image

## Abstract

**Objective:**

In radiation therapy, cancerous region segmentation in magnetic resonance images (MRI) is a critical step. For rectal cancer, the automatic segmentation of rectal tumors from an MRI is a great challenge. There are two main shortcomings in existing deep learning-based methods that lead to incorrect segmentation: 1) there are many organs surrounding the rectum, and the shape of some organs is similar to that of rectal tumors; 2) high-level features extracted by conventional neural networks often do not contain enough high-resolution information. Therefore, an improved U-Net segmentation network based on attention mechanisms is proposed to replace the traditional U-Net network.

**Methods:**

The overall framework of the proposed method is based on traditional U-Net. A ResNeSt module was added to extract the overall features, and a shape module was added after the encoder layer. We then combined the outputs of the shape module and the decoder to obtain the results. Moreover, the model used different types of attention mechanisms, so that the network learned information to improve segmentation accuracy.

**Results:**

We validated the effectiveness of the proposed method using 3773 2D MRI datasets from 304 patients. The results showed that the proposed method achieved 0.987, 0.946, 0.897, and 0.899 for Dice, MPA, MioU, and FWIoU, respectively; these values are significantly better than those of other existing methods.

**Conclusion:**

Due to time savings, the proposed method can help radiologists segment rectal tumors effectively and enable them to focus on patients whose cancerous regions are difficult for the network to segment.

**Significance:**

The proposed method can help doctors segment rectal tumors, thereby ensuring good diagnostic quality and accuracy.

## Introduction

Cancer is a worldwide problem that leads to death [1]. Rectal cancer [2] is one of the most common malignant cancers of the digestive tract. According to the GLOBOCAN 2020 Cancer Incidence and Mortality Assessment published by the International Agency for Research on Cancer [3], the incidence of rectal cancer ranks third of all kinds of cancers, and its mortality ranks second. With the impact of the aging population and unhealthy diet, rectal cancer tends to have the highest incidence and diagnostic rates. Generally, the period from normal intestinal tissue to canceration is very long, approximately 15–18 years, which proves that rectal cancer is difficult to find in early time [4] and results in a high rate of missed diagnosis of approximately 25%. Rectal cancer is detected in its middle and late stages, and its 5-year survival rate is 10%. Therefore, it is imperative to assist doctors in the diagnosis and treatment of rectal cancer with the help of emerging technologies [5] such as deep learning and neural networks.

**Fig. 1:**
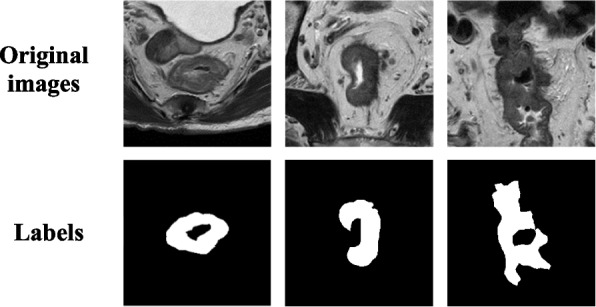
Typical examples of MR imaging of rectal cancer. Each MRI image of rectal cancer was from a different patient, and it was evident that the morphology of different rectal cancers varied greatly

Medical imaging is not only the prerequisite of medical image analysis but also an important way to determine a patient’s treatment plan. Currently, several imaging modalities are used for the preoperative assessment of rectal cancer, including colonoscopy, intrarectal ultrasound (EUS), and MR imaging. MR imaging has become the first choice in the diagnosis and treatment of rectal cancer because of its ability to provide patients with higher soft tissue contrast and because it has no radiation effects [6]. However, the automatic segmentation of colorectal cancer tumors from MRI images remains a great challenge, as tumor size and shape vary greatly depending on the pathological features and physical condition of different patients, as shown in Fig. 1. In addition, because of the large number of organs around the lesion area and the similarity in shape between some organs and rectal cancer tumors, the segmentation boundaries are not clear, which makes the segmentation of tumors more difficult.

Convolutional neural networks (CNNs) are the most representative deep learning algorithm, and they have achieved good results in the field of natural image analysis. Unlike traditional feature extraction methods, CNNs use an end-to-end working principle, i.e., they automatically extract task-related features from the input image and output corresponding results. Recently, with the increase in medical data and the improvement in computer power, the application of CNNs to the field of medical image analysis has received great attention from researchers and has become popular. Compared with manual segmentation, automatic segmentation [7] simplifies the workflow to rapidly process images without manual operations. Some automatic segmentation algorithms have been presented, such as the atlas-based model [8], random field model [9], and transformation model [10]. These model-based methods perform well in prediction, but they are not widely used because they often use several patient-specific parameters. The learning-based model [11] is mainly applied to the automatic segmentation of fine features. However, none of these approaches can deal with certain complex clinical problems because of the sensitive features of medical data and the instability of network structures. Structure-based models [12] achieve automatic segmentation by using the prior knowledge of the original data, but they tend to fail in segmenting rectal tumors because of the complex and variable shapes of these tumors. Recently, with the development of deep learning methods, they have performed well in the application of classification in medical fields [13, 14], which has led researchers to pay more attention to medical image segmentation [15, 16]. Many segmentation networks have emerged, including U-Net [17], DeepLabv3 [18], and SegNet [19]. Many CNNs for semantic segmentation have been applied to medical images, such as for the segmentation of the liver [20], heart [21], glands [22], and eyes [23]. The field of medical segmentation is dominated by approaches that are based on deep learning, among which U-Net has shown the strongest performance for the segmentation of medical images. However, U-Net has some drawbacks. For example, the structures of its encoders and decoders are simple; therefore, it has difficulty extracting deep features, and this leads to inaccurate segmentation.

In addition, the attention mechanism allows the CNN to focus on the region of interest while suppressing features in the background region, thereby improving the model classification performance. Jie et al. [24] developed a squeeze-and-excitation network (SENet) that determines the importance of each feature channel and assigns different weight coefficients to each channel. Ibtehaz et al. [25] and Park et al. [26] proposed a practical and lightweight bottleneck attention module (BAM) that can be integrated with any feed-forward CNN to allows the network to obtain a robust feature representation without adding many network parameters. Woo et al. [27] proposed a convolutional BAM (CBAM) that enables information interaction within space and between channels. Recently, researchers integrated the attention module into medical image segmentation networks to improve segmentation performance. For example, Ni et al. [28] enhanced the decoder with a newly designed attention module to emphasize the region of interest and improve the network representation of features. Yun et al. [29] proposed a dual attention module to focus attention on the location information of rectal tumors.

On the basis of the above findings, this study proposes a segmentation network based on the improved U-Net to achieve the automatic segmentation of rectal tumors from MRI. Our contributions are as follows:

1. Different network modules are combined in the traditional U-Net network to compose an advanced U-Net network that significantly improves segmentation performance.
2. Attention mechanisms are added to the improved U-Net to enable the network to focus on extracting the boundary features of rectal cancer during segmentation.

## Methodology

In this section, a new segmentation network based on U-Net is presented and its specific structure is explained.

Because connections across layers in the ResNet [30] can solve the problem of gradient disappearance, it is possible to build a deep neural network to improve the expressiveness of the model. In addition, CNNs have achieved great success in image recognition and segmentation in different application scenarios. Thus, based on the advantages of various neural networks, this study constructs a semantic segmentation network with layers and complex structures to meet the clinical needs of accurate cancerous region localization in rectal cancer images.

**Fig. 2:**
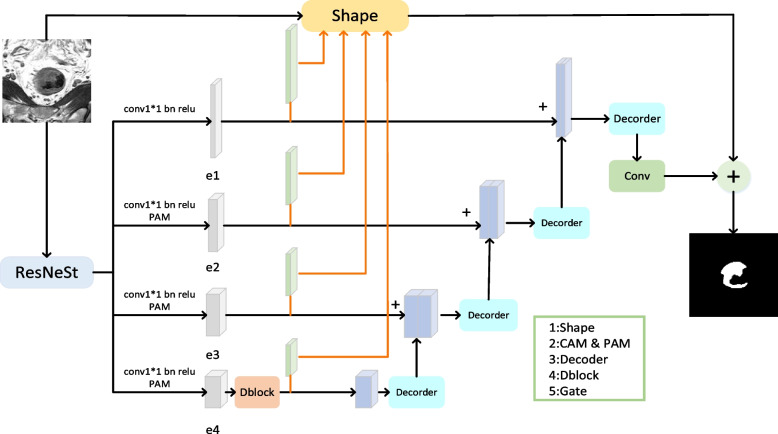
Overall network architecture. The input is a $256 \times 256$ 2D rectal MRI gray image, and the output is a $256 \times 256$ binary image that segments the cancerous region and background region based on pixels

The overall network architecture, which is inspired by the symmetrical structure of the classic U-Net network, is shown in Fig. 2. The ResNeSt [31] network is chosen as the encoder, which is mainly used to reduce the spatial dimensionality of the image and extract abstract features. The compression path comprises four groups of coding network blocks. The deconvolution layer is chosen as the decoder to realize upsampling, whose main function is to recover the details and positional information of the object. The extended path is also composed of four groups of blocks and establishes a fast connection with the four blocks of the compressed path. In addition to the backbone of the network, we also use position and channel attention modules (CAMs) to produce a distinguished feature representation, using $1\times 1$ convolution kernels to complete the channel transformation operation. Simultaneously, dilated convolution and cross-layer stitching are both used to realize the multiscale feature capture of different receptive fields. In the following, each component module and the related parameters of the network are described.

### ResNeSt module

**Fig. 3:**
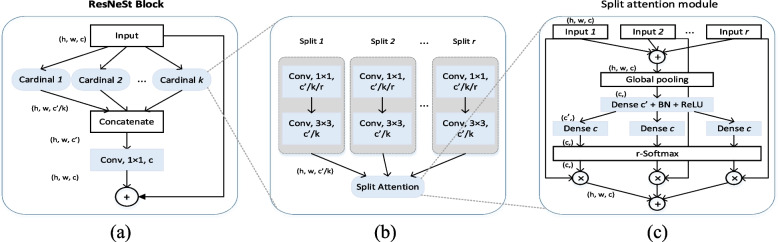
**a** Basic rest block structure is shown, with K cardinal groups of the same structure; **b** and **c** internal network structure of a single cardinal group is shown to intuitively explain the split attention working mechanism, where $c= C / K$

Split attention networks propose that due to the limited receptive field size and the lack of interaction between channels, the ResNeSt [31] network will perform well in image classification tasks, but it is not suitable for direct application to target detection, image segmentation, and other tasks. In contrast to [32–34], the network structures of split attention networks are designed and improved for specific tasks, while ResNeSt’s split attention blocks are backbone networks with general improvement functions and can be used as large-scale benchmarks for migration learning to apply cross-channel information to downstream tasks. As shown in Fig. 3, the feature graph with an input size of $H\times W\times C$ is divided into several groups, and the number of groups is determined by the cardinality hyperparameter. The number of splits in each group is determined by the hyperparameter radix. Taking a single grouping as an example, multiple splits are fuzed by element summation, and the global average pooling result is expressed as ${s}_{c}^{k}$, where $k\in \mathrm{1,2},...K$, $c\in \mathrm{1,2},...C/K$. The slice-weighted fusion means the *k-th* cardinality group can be expressed as follows:

1 $${V}_{c}^{k}={\sum }_{i=1}^{R}{a}_{i}^{k}(c){U}_{R(k-1)+i}$$

The slice weight ${a}_{i}^{k}(c)$ is given by the following formula, Eq. 2:

2 $${a}_{i}^{k}\left(c\right)=\left\{\begin{array}{ll}\frac{\mathit{exp}\left({g}_{i}^{c}\left({s}^{k}\right)\right)}{{\sum }_{j=0}^{R}\mathit{exp}\left({g}_{i}^{c}\left({s}^{k}\right)\right)} & if\,R>1 \\ \frac{1}{1+\mathit{exp}\left(-{g}_{i}^{c}\left({s}^{k}\right)\right)} &if\,R=1\end{array}\right.$$

The cardinality groups are spliced along the channel dimension: $V=Concat\{{V}^{1},{V}^{2},...{V}^{k}\}$, and they perform a cross-layer connection summation operation that is similar to the standard residual block: $Y=V+f(X)$. The encoder adopts the ResNeSt200 basic model and its pretraining parameters. Super parameters $K=1$ and $R=2$ ensure a good trade-off between speed, accuracy, and memory usage.

### DBlock and decoder

**Fig. 4:**
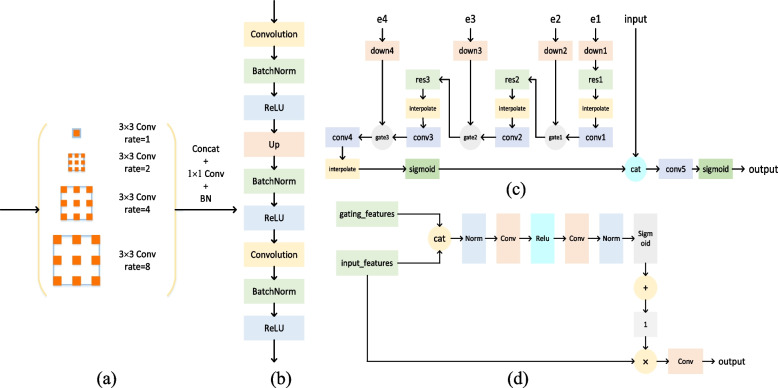
**a** Dblock module: schematic diagram of multiscale information acquisition based on dilated convolution; **b** Decoder module: schematic diagram of the upsampling process based on deconvolution; **c** e1, e2, e3, and e4 obtain an output of 1 × 256 × 256 through shape transformation and fusion with the output feature map of the decoder to obtain the final segmentation prediction result based on pixels; **d** gate module: schematic of feature map fusion based on spatial weights

The output size of the fourth group of ResNeSt blocks is $1024 \times 8 \times 8$ and a $3 \times 3$ convolutional kernel is used to realize multiscale transformation whose dilation rates are 1, 2, 4, and 8. The advantage of dilated convolution is that it increases the receptive field without pooling the loss information, so that each convolution output contains a wide range of information. Multiscale context information is conducive to meeting the segmentation requirements of large and small objects at the same time. As shown in Fig. 4a, the input performs four groups of convolution transformations with different dilation rates and concatenates with the original input to obtain the output of $512 \times 8 \times 8$. Then, the number of channels is restored to 1024 through the convolution operation.

The output of image segmentation prediction is pixel-wise; therefore, the smaller image size after convolution and pooling should be upsampled to the original image size for prediction. Upsampling generally adopts a deconvolution operation. The previous dilated convolution operation makes each pixel prediction based on the larger receptive field information. As shown in Fig. 4b, the lowest output is decoded through the deconvolution decoding process to restore the size from 1024 × 8 × 8 to 512 × 16 × 16. The steps of the three sampling processes are the same. Before decoding, the input establishes a quick connection with the corresponding block output of the compression path for element-wise summation.

### Shape module

In essence, shape transformation still involves splicing feature maps at different layers. Its main advantage is that it fuzes features of different scales, dimensions, and stages in the output layer so that this layer contains richer information and improves the segmentation accuracy. As shown in Fig. 4c, the input image is spliced with the four outputs, e1, e2, e3, and e4, in the coding stage and then fuzed with the decoder output to obtain the final output result. The sizes of e1, e2, e3, and e4 are smaller than $256\times 256$, so it is necessary to use the interpolate layer to realize the upsampling operation of bilinear interpolation and to use the $1\times 1$ convolution kernel to realize the compression channel operation.

The gate module is used for the fusion of e1, e2, e3, and e4, as shown in Fig. 4d. Taking $e4+e3$ as an example, the result of e4 after the compression channel is treated as an input feature and is represented by $I$, and the result of e3 after the compression channel is treated as a gating feature and is represented by $G$. Gating and input features are spliced in the channel dimensions and transformed into a spatial weight matrix, represented by $W$. The final output of the gate module is the result of the input features corrected on the basis of spatial weight information, represented by ${I}^\prime$. The formula is expressed as follows:

3 $${I}^\prime=I(W+1)$$

### Attention mechanism: PAM and CAM

**Fig. 5:**
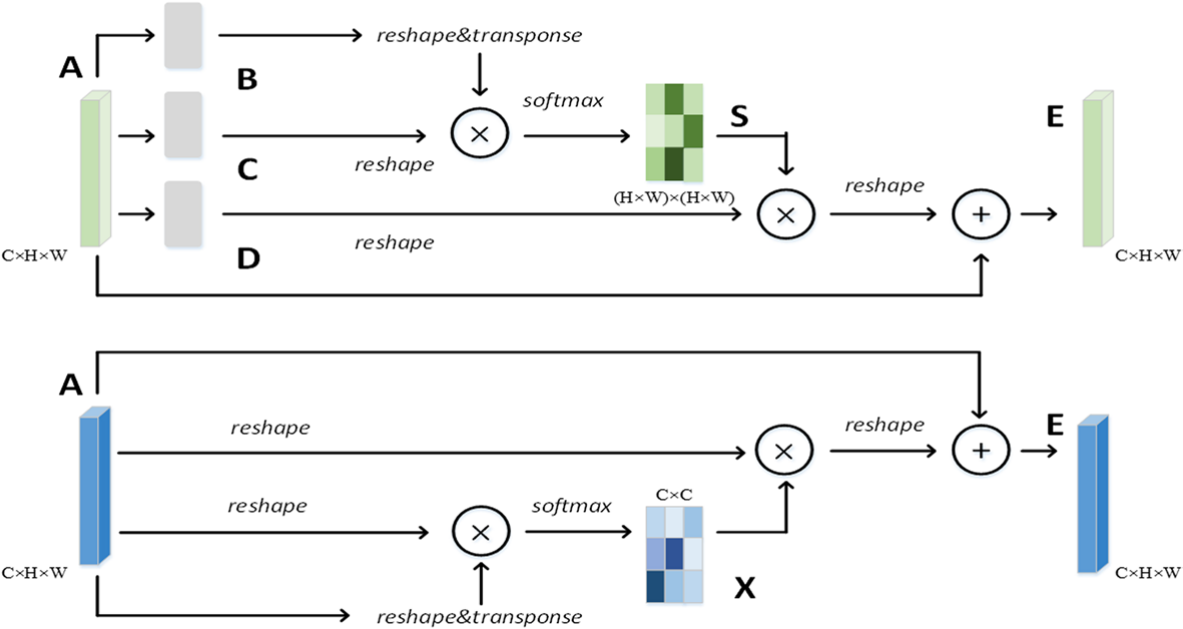
PAM and CAM are shown in figures (**a**) and (**b**)

The position attention module (PAM) and CAM [35] improve the model accuracy of semantic segmentation tasks effectively and have good universality, so they are used in this study. Figure 5 shows a structure diagram of the PAM and CAM.

PAM uses the association between any two features to enhance the expression of their respective features. It has a global context, and local features are encoded by the broader context information. In addition, similar semantic features gain from each other, thereby improving intraclass compactness and semantic consistency. The expression is given by Eqs. 4 and 5:

4 $${s}_{ji}=\frac{exp({B}_{i}\cdot {C}_{j})}{{\sum }_{i=1}^{N}exp({B}_{i}\cdot {C}_{j})}$$

5 $${E}_{j}=\alpha {\sum }_{i=1}^{N}({s}_{ji}{D}_{i})+{A}_{j}$$

where ${s}_{ji}$ is the location-based correlation coefficient matrix, which measures the impact of the $ith$ spatial location on the $jth$ spatial location. To meet the requirements for matrix multiplication, ${B}_{i}$ and ${C}_{j}$ perform flattening transformation and transposition operations on the $H$ and $W$ dimensions. The value of the final output $E$ in the position of $j$ is obtained by adding the weighted sum of all position features based on the correlation coefficient and the original feature value. $\alpha$ is the scale parameter that controls the degree to which weighted features correct the original features.

CAM uses the association between any two channel features to enhance the expression of their respective features. By mining the interdependence between channel mappings, strongly related channels are emphasized and the feature representation of specific semantics is improved. The biggest difference between CAM and squeeze-and-excitation (SE) is that the channel correlation calculation uses the information from all elements in the channel instead of their global average pooling results. The expression is given by Eqs. 6 and 7:

6 $${x}_{ji}=\frac{exp({A}_{i}\cdot {A}_{j})}{{\sum }_{i=1}^{C}exp({A}_{i}\cdot {A}_{j})}$$

7 $${E}_{j}=\beta {\sum }_{i=1}^{C}({x}_{ji}{A}_{i})+{A}_{j},$$

where ${x}_{ji}$ is the correlation coefficient matrix based on the channel, which measures the influence of the $ith$ feature channel on the $jth$ feature channel. To meet the requirements of matrix multiplication, ${A}_{i}$ and ${A}_{j}$ perform flattening transformation and transposition operations on the $H$ and $W$ dimensions. The value of the final output $E$ in the position of $j$ is obtained by the addition of the weighted sum value of all channel features based on the correlation coefficient and the original feature value. $\beta$ is the scale parameter that controls the degree to which the weighted features correct the original features.

### Loss function

The loss function used in this study is the Dice loss, which is defined as in Eq. 8:

8 $$dice=\frac{2\left|G\cap P\right|}{\left|G\right|+\left|P\right|}$$

where $G$ represents the tumor region in the label image and $P$ represents the tumor region in the predicted image.

**Fig. 6:**
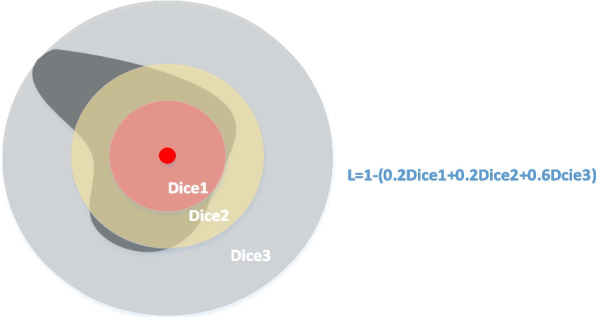
Schematic of the loss calculation based on centroid interval division

First, the centroid coordinates and the farthest tumor pixel coordinates of the colorectal tumor area should be calculated. Second, the tumor area is divided into three equal rings, called the inner, middle and outer rings. The Dice for each ring is then calculated. Finally, the loss function is defined as in Eq. 9:

9 $$loss=1-\left({k}_{1}dic{e}_{1}+{k}_{2}dic{e}_{2}+{k}_{3}dic{e}_{3}\right),$$

where $dic{e}_{i}$ represents the interval loss of a circular area from the inside out, and ${k}_{i}$ is the balance weight used to balance the relationship between the three losses. Due to the irregularity and nonconnectivity of tumors, it is difficult to segment pixels in the area of the tumor edge. Increasing the weight coefficient $dic{e}_{3}$ appropriately helps the model focus on learning the segmentation of challenging samples. Figure 6 shows the calculation principle of the loss function, where the value of ${k}_{i}$ is a random example.

## Experimental preparation

### Data

The data used in this study were obtained from Shanxi Provincial Cancer Hospital, and they include 3773 2D MR rectal cancer images obtained from 304 patients with the T2WI sequence. All MR rectal cancer images were collected using a 1.5-T GE Signa MR355 scanner. The MR images were converted to grayscale images with pixel values in the range of [0, 255]. The contents of the MR images were labeled by experienced radiologists. 150 images were randomly selected from 3773 2D MR images as the test set, and the remaining images were divided into the training and validation sets in a 9:1 ratio.

### Data enhancement

The Albumentations package was used for data enhancement, which included horizontal flip, vertical flip, random rotation by 90°, grid transformation, elastic transformation, and random gamma enhancement.

### Implementation details

The overall network was programed using Python 3.6 and the PyTorch framework. The experiments were performed on a workstation equipped with an Intel Core i9-10900X CPU, four 32 GB RAM, GPUs, two GeForce RTX 3090 Turbo graphics cards, and the Ubuntu 18.04 operating system. We used the MR rectal image dataset to evaluate our method and employed a ten-fold cross-validation approach to validate the generalization performance of the proposed model. In the experiments, we used the AdamW optimizer to optimize the parameters of the model at training time. Empirically, we set the model start parameters as follows: the initial learning rate was 1e − 4, weight_decay was 1e − 5, and batch_size was 8. Furthermore, we used cosine annealing as the learning rate adjustment function. Moreover, the image size of the input network was 256 × 256.

### Evaluation metrics

#### Dice

Dice is a measure of the similarity between the two sets. It is used to measure the similarity between network segmentation results and the gold standard in the field of image segmentation, and is defined as follows:

10 $$dice=\frac{2\left|G\cap P\right|}{\left|G\right|+\left|P\right|}$$

where $G$ represents the tumor region in the label image and $P$ represents the tumor region in the predicted image.

#### Mean pixel accuracy (MPA)

MPA is the average ratio of the number of correct classification pixels in each category to the number of all pixels in that category, as defined by Eq. 11:

11 $$MPA=\frac{1}{k+1}{\sum }_{i=0}^{k}\frac{{p}_{ii}}{{\sum }_{j=0}^{k}{p}_{ij}}$$

where ${p}_{i,j}$ denotes the number of true values i that are predicted to be j, and k + 1 is the number of categories (including the empty categories).

#### Mean intersection over union (MIoU)

MIoU is the ratio of the intersection of the true and predicted values to the union of the true and predicted values, as defined by Eq. 12:

12 $$MIoU=\frac{TP}{FP+FN+TP}$$

where TP, FP, TN, and FN represent the number of true positives, false positives, true negatives, and false negatives, respectively.

#### Frequency-weighted intersection over union (FWIoU)

FWIoU is the weighted sum of the IoU of each category, where the weights are calculated based on the frequency of each category. It is defined as in Eq. 13:

13 $$FWIoU=\frac{1}{{\sum }_{i=0}^{k}{\sum }_{j=0}^{k}{p}_{ij}}{\sum }_{i=0}^{k}\frac{{p}_{ii}{\sum }_{j=0}^{k}{p}_{ij}}{{\sum }_{j=0}^{k}{p}_{ij}+{\sum }_{j=0}^{k}{p}_{ji}-{p}_{ii}}$$

where $p_{i,j}$ denotes the number of true values i that are predicted to be j, and k + 1 is the number of categories (including empty categories).

## Experimental results and visualization

Two types of experiments are presented in this section. The experimental results and visual information are presented more intuitively.

### Ablation experiments

To evaluate the influence of the components in the improved U-Net network, ablation experiments were performed by adding, removing, or replacing those components. To achieve a fair comparison, all ablation experiments used the control variable method.

#### Impact of each added module

In this section, the impacts of each module in the proposed U-Net network on the segmentation results are compared, as shown in Table 1a.

**Table 1:** Comparison results

**Method**	**Dice**	**MAP**	**MIoU**	**FWIoU**
**(a) Comparison results for each component**				
Without ResNeSt	0.923	0.825	0.776	0.781
Without shape	0.901	0.811	0.734	0.740
Without PAM&CAM	0.958	0.786	0.803	0.791
**Proposed U-Net**	**0.987**	**0.946**	**0.897**	**0.899**
**(b) Comparison results for different attention mechanisms**				
With SE	0.935	0.755	0.645	0.611
With GC	0.949	0.809	0.774	0.740
With CBAM	0.961	0.902	0.812	0.827
**Proposed U-Net**	**0.987**	**0.946**	**0.897**	**0.899**
**(c) Comparison of the different backbones used in the proposed U-Net network**				
ResNet34	0.935	0.665	0.398	0.423
SEResNeXt50	0.951	0.805	0.734	0.752
SENet-154	0.958	0.911	0.860	0.854
**ResNeSt**	**0.987**	**0.946**	**0.897**	**0.899**
**(d) Effect of the gate module**				
Without a gate module	0.973	0.922	0.855	0.861
**With the gate module**	**0.987**	**0.946**	**0.897**	**0.899**
**(e) Results of comparison with existing advanced models**				
DeepLabv3 [18]	0.938	0.745	0.570	0.566
U-Net++ [36]	0.943	0.811	0.681	0.682
U-Net+++ [37]	0.925	0.707	0.550	0.554
GSCNN [38]	0.910	0.602	0.419	0.510
ERFNet [39]	0.946	0.843	0.473	0.473
ET-Net [40]	0.927	0.862	0.689	0.784
**Proposed U-Net**	**0.987**	**0.946**	**0.897**	**0.899**

Each component was removed separately to carry out the experiments, and the experimental results were compared. First, we tested the network when the ResNeSt module before the encoders were removed, and the results showed that Dice, MPA, MIoU, and FWIoU were 0.923, 0.825, 0.676, and 0.681, respectively. Second, the Shape module between the output of the encoder and the final output was removed, and the results showed Dice, MPA, MIoU, and FWIoU values of 0.901, 0.811, 0.634, and 0.640, respectively. Finally, the attention mechanisms (PAM and CAM) in the improved U-Net were removed, and the Dice, MPA, MIoU, and FWIoU values were 0.958, 0.786, 0.603, and 0.611, respectively. The results showed that the proposed network showed significant improvement. Consequently, all three components were significant when added to the construction of the new U-Net network.

#### Impacts of different attention mechanisms

Different attention mechanisms were added to the network to evaluate their effects, as shown in Table 1b.

First, the SE method was selected, and the results achieved Dice, MPA, MIoU, and FWIoU values of 0.935, 0.755, 0.645, and 0.611, respectively. Second, we selected the global context (GC) to obtain results for Dice, MPA, MIoU, and FWIoU values of 0.949, 0.809, 0.774, and 0.740, respectively. Finally, we used the CBAM to get the results for Dice, MPA, MIoU, and FWIoU of 0.961, 0.902, 0.812, and 0.827, respectively. All of the above attention mechanisms showed poorer results than did PAM or CAM. Therefore, the PAM and CAM attention mechanisms were included in the network.

#### Impacts of various backbones

The replacement of the encoders with different backbones was compared, and the results are shown in Table 1c.

The results show that when the ResNeSt model is selected as the encoder, the Dice, MPA, MIoU, and FWIoU reach their highest values of 0.987, 0.946, 0.897, and 0.899, respectively, which indicate obvious advantages over other backbones. Therefore, the ResNeSt model was adopted in the improved U-Net.

#### Impacts of the gate module

The gate module was introduced into the proposed U-Net. Its effect is presented in Table 1d.

It is obvious that when the gate module is included, Dice, MPA, MIoU, and FWIoU improve by 0.014, 0.024, 0.042, and 0.038, respectively. Therefore, the addition of a gate module is necessary to improve the performance of the proposed segmentation networks.

### Comparison with existing advanced models

In this study, an improved U-Net segmentation network based on attention mechanisms is proposed. In this section, the differences in performance between the proposed network and the existing advanced models are compared, and are shown in Table 1e.

The results of the experiments are summarized in Table 1e. The results in the bottom row are the best results obtained on the test set, which show that the proposed method is significantly better than all the other networks.

### Visualization results

**Fig. 7:**
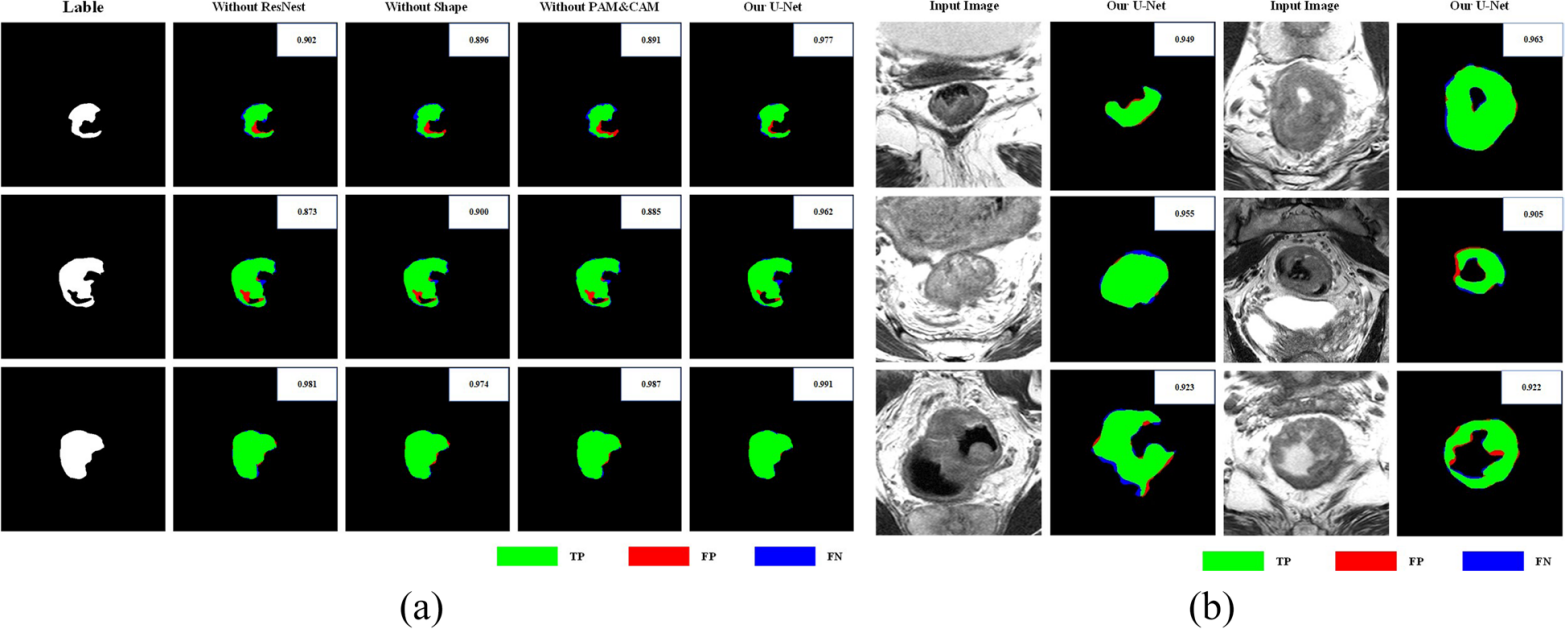
**a** Visualization of comparison results for the impact of each component on the improved U-Net network. In this figure, true positive is shown in green, false positive is represented in red, and false negative is in blue. **b** Comparison of input images and output results. In this figure, true positives are shown in green, false positives are represented in red, and false negatives are shown in blue

The comparison results related to the impact of each component to improve the proposed U-Net network are shown in Fig. 7a.

In this figure, the Dice value for each image is shown in the upper-right corner. In addition, the different cases are presented in different colors, where the true positive is in green, the false positive is in red, and the false negative is in blue. The results show that the improved U-Net performed best.

Figure 7b shows the output results compared with the original input images, where green represents true positive, red represents false positive, and blue represents false negative. According to these results, the improved U-Net proposed in this study showed good performance in rectal cancer segmentation for 2D MR images.

## Conclusion

In this study, an improved U-Net segmentation network based on an attention mechanism is proposed. The segmentation performance was improved by adding different training modules to the traditional U-Net, including the ResNeSt, Shape, gate, and visual mechanism modules. This method can effectively address the challenge of rectal tumors being surrounded by many similar organs, as well as the problem of significant changes in cancer shape, and makes it easy to segment rectal tumors from the original MR images. The results showed that the proposed method achieves better results than do the other methods. Furthermore, the proposed method can be used to segment other medical images. In the future, methods in the 3D segmentation field should be studied to meet clinical requirements according to the clinical information.

## Data Availability

The datasets generated and analyzed during the current study are not publicly available due to the limitations in hospital confidentiality agreements, but they are available from the corresponding author upon reasonable request.

## Abbreviations

MRI: Magnetic resonance images
CNN: Convolutional neural network
SENet: Squeeze-and-excitation network
BAM: Bottleneck attention module
CBAM: Convolutional bottleneck attention module
CAM: Channel attention module
PAM: Position attention module
MPA: Mean pixel accuracy
MioU: Mean intersection over union
FWIoU: Frequency-weighted intersection over union
GC: Global context
